# Conceptualizing National Advisory Boards in primary care research: application to the Two in One HIV and COVID screening and testing model

**DOI:** 10.1017/S1463423624000100

**Published:** 2024-05-09

**Authors:** Maranda C. Ward, Paloma Delgado Setien, Abigail Konopasky, Donaldson F. Conserve

**Affiliations:** 1 Department of Clinical Research and Leadership, School of Medicine and Health Sciences, George Washington University School of Medicine and Health Sciences, Washington, DC, USA; 2 The Milken Institute School of Public Health at The George Washington University, Washington, DC, USA; 3 George Washington University School of Medicine and Health Sciences, Washington, DC, USA; 4 Department of Medical Education, Geisel School of Medicine at Dartmouth, Hanover, NH, USA

**Keywords:** advisory boards, culturally responsive communication, research

## Abstract

The authors report on their development of a National Advisory Board (NAB) to guide a funded project: Two in One: HIV + COVID-19 Screening and Testing Model. This project aimed to improve primary care practitioners’ capacity to routinize HIV, PrEP/PEP, and COVID-19 vaccine screenings for all their patients while relying on culturally responsive communication with their minoritized patients. To approach their monumental research and education tasks, they created a NAB, drawing from the literature on advisory boards to (a) promote board member engagement and (b) progress successfully through the six stages suggested for successful advisory boards. A midpoint survey and final focus groups with NAB members indicated mixed levels of engagement, a sense of time and work being valued, and pride in the media and academic reach of the project. The authors offer considerations for others considering forming a NAB to guide primary care research and interventions.

## Introduction

In the United States, HIV and COVID-19 disproportionately affect minoritized populations (Earnshaw *et al.*, 2013; Khanijahani *et al.*, 2021). Yet, many primary care practitioners (PCPs) do not have the capacity to meet the dynamic needs of these patient populations. To address this, we sought funding to develop training to expand PCPs’ capacity to make culturally responsive recommendations for HIV and COVID-19 vaccination screenings. To increase trainings’ efficacy and value, we involved a variety of experts. As we looked to convene a deliberately curated National Advisory Board (NAB), we found some guidance on structures and processes, but no case studies of their use in primary care research to guide us. In this Short Report, we share our experiences applying two frameworks for scientific advisory committees to our national training efforts (Hoffman *et al.*, 2018; Courtney *et al.*, 2021).

## Background

When we began, the COVID-19 pandemic had laid bare structural inequities making racial, ethnic, sexual, and gender-minoritized communities more vulnerable to severe illness and death (Ward, 2023a). Yet, media narratives laid the blame on these communities, dubbing them ‘vaccine-hesitant’, despite overwhelming evidence that, for instance, Asian, Hispanic, and Native Americans had *higher* vaccination rates than White populations (Chowkwanyun and Reed, 2020; Satcher Health Leadership Institute, 2021). Meanwhile, the ongoing HIV pandemic continued to disproportionately affect these *same* communities, illuminating the role poverty and disparate access to healthcare and quality housing play in poor health outcomes (Grey, 2021). These structural inequities are exacerbated by generational trauma, medical mistrust, racism, homophobia, transphobia, and ongoing discrimination contributing to misinformation, fear, and stigma around the prevention, spread, and treatment of both viruses (Kendi, 2020). Meanwhile, we witnessed a sole focus on COVID-19 prevention contributing to less attention to HIV prevention.

Thus, we created the ‘Two in One: HIV + COVID-19 Screening and Testing Model’ to improve PCPs’ capacity to routinize HIV, pre-exposure prophylaxis and post-exposure prophylaxis (PrEP/PEP), and COVID-19 vaccine screenings for all their patients while relying on culturally responsive communication with their minoritized patients (Ward, 2023b). PCPs must be adequately prepared to address the ongoing threats to racial and health equity (*Healthy People 2030*, no date), yet many are not (Hoover *et al.*, 2020). For instance, despite the 2006 CDC recommendation for opt-out HIV testing for those between the ages of 13 and 64 (Centers for Disease Control and Prevention, 2022), many PCPs continue to test sporadically and only based on perceived risk, even while testing in community health centers and emergency departments remains on the rise (Hoover *et al.*, 2020).

To approach our monumental task – researching the experiences of minoritized patients with COVID-19 and HIV primary care, developing nine national webinar training based on this research, and creating a permanent online course offering Continuing Medical Education credit – we decided to create a NAB. We looked to the literature on advisory boards but found few scholars publishing on this critical part of the research process: most were drawn from local communities rather than a national one, and none of it was in primary care research. The literature we did find, however, argued for the important role of advisory boards. The types ranged from community advisory boards, Scientific Advisory Boards, and Scientific Advisory Committees to Expert Advisory Boards (we refer throughout to ‘NABs’). For instance, Courtney and colleagues call advisory boards a ‘critical instrument of governance’ (Courtney *et al.*, 2021: p.254). They provide the expertise of experience, both positive and negative, and can be used to leverage the cooperation of the community involved. They are also ideal problem-solving tools because there are no predetermined formal procedures constraining them, its members can focus directly and solely on the issues they were created to address (Reiter, 2003).

Yet, advisory boards are not guaranteed to be successful. They must be deliberately structured and its members need support. Rapattoni (2008) noted the importance of frequent meetings to maintain retention, of creating a safe environment in meetings where members can speak freely without criticism, and of holding members accountable to the project’s aims to ensure the board’s integrity that it is also important for NAB members to be engaged in some of the work of the project to increase a sense of buy-in. Further, in order to best support the project, board members need training regarding their specific functions on the board (Ripley, Cummings, and Lockett, 2012).

Hoffman and colleagues offer a framework to generate successful boards (and, in turn, successful projects) that involves 6 stages: (1) Initial establishment, (2) Member selection, (3) Advice Generation, (4) Advice Delivery, (5) Advice Implementation, and (6) Monitoring and Evaluation, providing guidelines and clear *determinants of effectiveness* for each of these stages (Hoffman *et al.*, 2018). Additionally, Courtney and colleagues provide critical suggestions for engaging NAB members (Courtney *et al.*, 2021). The purpose of this Short Report is to demonstrate how our application of Hoffman *et al.*’s and Courtney *et al.*’s approaches strengthened our work and the implications this has for others doing research in primary care.

## Approach

We followed Hoffman and colleagues’ framework through each of the six stages as we developed, worked with, and evaluated our NAB. First, to establish the committee, the core research team consisting of the primary investigator (PI) and all co-investigators (Co-I’s) met to talk about project needs and challenges. This group determined that we needed expertise in the following areas: HIV and COVID-19 patient populations, primary care, HBCU pipeline programs, minoritized populations, racial/health equity, and student/trainee populations. Moreover, we wanted lived expertise from these communities of interest. Based on our required areas of expertise, we sent invitations for participation in the NAB to health professionals across the United States.

Second, after reaching out to 29 potential members, we selected our NAB. The final board was established with 11 participating members, composed of one gender non-conforming person, five women, and five men with expertise across our desired areas. Per Hoffman *et al.* ’s suggestion, we took a democratic approach to committee selection (the core team decided on membership together) and carefully managed conflict of interest (eg, some board members turned down the honorarium since they were part of the federal government).

Third, we began the process of generating advice. The NAB met five times across the grant period (see Table 1), each session strategically scheduled at a key juncture in the project when the core team needed advice. As Hoffman *et al.* suggest, we offered clear guidance for making decisions. We sent introductory emails out with expectations for the board (eg, attending all meetings, and brief meeting preparation in some cases). Further, we had structured agendas for each meeting and shared minutes of meetings with all members. Cognizant of our NAB members’ limited time, we presented project updates in easily accessible and brief presentations, only focusing on the evidence they would need to make the decisions we were asking for at that point in the grant. Rather than a formal vote, we asked each NAB member to weigh in separately, as consensus methods can stifle some voices.

**Table 1.** National advisory board meeting structure and advice application across 18-month Two in One project

Project month	Key advice needed	Structure of meeting	Application of advice
3	Strategies for recruiting research participants	– Group introductions – Project overview summary – Expectations as NAB members – Call for research recruitment help	Reached out to newly identified networks to recruit participants
6	Opinions on proposed training topics and additional topic suggestions	– Presentation of project updates – Presentation of research findings – Discussion of proposed topics (based on findings)	Revised topic list and finalized training schedule
9	Input on proposed policy white papers; mid-project feedback via survey	– Presentation of project updates – Policy advice sessions in breakout rooms – Administering NAB survey	Revised the three policy white papers; greatly expanded White paper distribution list
14	Suggestions for academic dissemination; guidance advertising trainings and course	– Presentation of project updates – Discussion of dissemination – Discussion of monthly trainings and permanent online course	Expanded list of conferences and journals for dissemination; revised advertising strategy for trainings and course
16	Feedback on NAB experience	– Presentation of project updates – Feedback sessions in breakout rooms	Made changes to NAB for next project proposal

Fourth, advice about our trainings was delivered through the NAB meetings themselves, which were recorded and transcribed. This gave team members access not only to members’ opinions and decisions but to the dialogic *process* that they used to reach them. Further, the PI communicated regularly with NAB board members over email and, when requested, individual meetings. These emails and meetings not only offered further clarification of member positions but were also opportunities for them to share resources (eg, literature, similar programs, contacts in the field), for the PI to build relationships, and to develop writing partnerships. All data (recordings, transcriptions, and emails) were kept private and were only accessible by the PIs, Co-I’s, and the research assistants.

Fifth, advice implementation was dialogic and emergent. Since team members were often weighing different opinions and resources, the team used its weekly meetings to debrief and discuss NAB advice and team impressions of its utility, feasibility, and importance. Implementation proceeded in an emergent fashion: after applying each piece of advice in the research or trainings, the team discussed its success and next steps for building on the results, often bringing back other NAB advice to take a different approach.

Sixth, we monitored the work of the NAB across the 18-month project, seeking both NAB and core team members’ experiences of the work and how it could be improved. We report on the methods and results of this evaluation below.

Finally, following Courtney and colleagues, we attempted throughout the process to engage NAB members in the work of the project. We partnered with them on writing policy, research, and commentary pieces and co-presented with them at conferences.

## Evaluation methods

To evaluate the effectiveness of the approach above (after receiving an IRB ‘exempt’ status), we gathered metrics on engagement throughout the program period such as the number of NAB members who attended meetings, contributed to publications, and co-authored presentations. We also anonymously surveyed NAB members about their experiences at the project midpoint, and gathered focus group feedback at the end. The electronic survey included nine Likert-scale items that cut across four areas including advice generation, monitoring and evaluation, initial establishment, and advice delivery. The focus group took place as breakouts during a virtual meeting and included four open-ended questions such as, ‘What was the most meaningful way you felt you contributed to our success?’ We analyzed the survey using descriptive statistics and analyzed the focus groups relying on themes.

## Results

Engagement with some board members was high: For instance, the core team co-presented research with two members, published articles with four members, and involved ten members directly in creation of our training materials (either recording one of our training or recruitment videos or developing content for the online course). Yet several NAB members did not respond to our invitations, only communicating during scheduled meetings and even missing some of those.

On the mid-project survey (response rate: 91%, 10 respondents/11, after accounting for one person who seems to have responded twice), most participants (91%–100%) agreed or strongly agreed that their expertise was valued and they had time and space to share their advice. One participant disagreed but did not leave open-ended comments explaining their experience.

In focus groups (of 3–4 members in three small groups), 11 NAB members were proud of the media and academic reach of the project and found participation in publications and conferences particularly personally meaningful. Members suggested even greater future engagement, including a retreat, more individual consultations, and engaging every member in at least one publication.

## Discussion

Following existing frameworks for advisory boards, we not only generated actionable and useful advice for our NAB but also engaged with board members to create some ongoing research and advocacy partnerships. We found this *reciprocity* of engagement important so that, like the work itself, our NAB interactions reflected our values of social justice and community engagement.

We offer several promising practices. First, while board members and the board leader participated in meetings, we struggled to follow Courtney *et al.*’s advice for deeper engagement due to their varying capacity and professional commitments (Courtney *et al.*, 2021). Yet, even their participation in meetings was critical, so as we continue this research, we plan to *offer* deeper engagement to each member, but not require it, and to identify a board member to support the PI.

Second, we were not able to identify the single board member who was dissatisfied. In future projects, we will have one-on-one meetings with NAB members at the midpoint to diagnose potential issues while we can still ameliorate them.

Finally, while we recognize that many doing research in primary care do not have the funding to compensate NAB members as we did, we felt this was a critical part of their work. We believe fair compensation is an equity issue and urge research funders to consider supporting NABs.

For others considering using NABs to guide primary care research, we recommend both Courtney *et al.*’s emphasis on engagement^3^ and Hoffman *et al.*’s framework (Hoffman *et al.*, 2018; Courtney *et al.*, 2021). As the latter note, ‘good evidence alone is an insufficient basis for good policymaking…science cannot operate in a silo and must take into consideration the larger normative concerns facing policymakers’ (Hoffman *et al.*, 2018: p.1). Engaging a NAB in this important effort helped us to step outside of this silo and create community and expert-informed, relevant, timely, and useful trainings to combat structural inequities in COVID-19 and HIV prevention.
